# Reshaping Parental Ethnotheories of Dutch‐Moroccan Immigrant Parents in the Netherlands: Networking in Multiple Worlds

**DOI:** 10.1002/cad.20339

**Published:** 2020-05-19

**Authors:** Mariëtte de Haan, Marije Koeman, Micha de Winter

**Affiliations:** ^1^ Utrecht University; ^2^ Koninglijke Aurisgroep

## Abstract

Ethnotheories of immigrant parents residing in the Netherlands are reshaped in response to the multiple and diverse educational practices they come in contact with after migration. Network analyses of “parenting relationships” of first generation Dutch‐Moroccan parents living in the Netherlands show that they borrow from diverse resources including professionals and nonprofessionals in their construction of new ethnotheories. Through media as well as through interacting with family in their country of origin, with same‐generation peers in the Netherlands, and with Dutch professionals and neighbors, these mothers develop “modern” notions of parenting such as stimulating child independence, while also using building blocks from traditional practices such as respect for the elderly. Individual variability is evident in parents’ processes of adaptation, with some parents seeming stuck between these alternative and seemingly contradictory practices and ideas while others learn to use them to position themselves optimally in their multi‐ethnic environment. The paper argues that recognizing these parents as creative producers of their own solutions, and becoming conscious of their self‐made support networks and the resources they provide, can offer professionals and policy makers a new paradigm for the design of social services and support for immigrant parents.

## Introduction

In the literature on postmigration parenting beliefs and practices, length of stay and number of generations born in the new country are often seen as indicators of a gradual change toward mainstream practices and values. The transformation that is a result of that change is often explained with the concept of acculturation. In acculturation theories, the degree to which migrants hold on to the country of origin (or stay “the same”) is set against the degree to which they seek contact with the host culture (or transform into the cultural system of the other), even if these are not seen as mutually exclusive (Bornstein & Cote, 2004; Schwartz, Montgomery, & Briones, 2006). Transformation in this case is seen as a matter of degree of adaptation or distancing from a particular cultural practice. In addition, cultural practices are seen as closed, relatively fixed systems that are not subject to change. Interactivity between cultural systems such as the home culture and the host culture, and how this can play a role in these transformative processes, are not acknowledged, or at least do not play a major role. The fact that individuals might for instance, combine elements of several cultural practices and create new practices, and that this again might contribute to the transformation of these practices “themselves” is often ignored. This limitation holds despite the extensive debate about the fact that immigrants can both hold on to certain aspect of their culture and at the same time adopt new values and practices (Berry, 1980; Gibson, 1987; Joe, 1994; Kwak & Berry, 2001; Suàrez‐Orozco & Suàrez‐Orozco, 2001) and about how acculturation is multivariate and modular and can develop at a different rate and to different degrees for different domains of acculturation depending on the nature of the contact with mainstream cultures (Bornstein & Cote, 2004).

Acknowledging that in a globalized world, cultural confrontations are much more complex than these prior theories assume, a growing body of literature has focused on the construction of hybrid parenting practices that arise within so called “contact zones” among multiple traditions of parenting (Reese, 2002; Roer‐Strier et al., 2005; Bacallao & Smokowski, 2007; Cook, & Waite; 2016; Bejenaru, 2018; Perreira, Chapman, & Stein, 2006; van Beurden & de Haan, 2019). “Hybrid” stands here for new practices that grow out of the coming together of two or more older ones. The concept of a contact zone, defined as those spaces which allow cultural heritages to meet, clash and struggle with each other (Pratt, 1991) and the idea of “cultural translation” (Papastergiadis, 2000), which takes into account the fact that cultural systems undergo a qualitative change after such a clash, turns the focus to recognition that in migration, particular cultural traditions of parenting come into existence which cannot be captured in pregiven categories (de Haan, 2011). For example, Kibria (1993) shows how Vietnamese immigrant families in the United States struggle with being in between the Vietnamese and American cultural spheres and finally are able to develop new strategies to combine elements of both. She describes how Vietnamese families have found the traditional, cooperative and patriarchal family system to be an effective means to run small businesses, which has helped them survive economically. However, as adolescents express a growing discontent with the hierarchical aspect of this traditional family model, the model is challenged and bent toward more egalitarian parent–child relationships. The coming together of “old” and “new” elements, as well as how these were confronted and challenged led to the development of a successful new family business model. This new model was formed both with the help of the cooperative family model from their country of origin, as well as with the help of new models of family relationships they adopted from the United States. In another more recent study, Bacallao and Smokowski (2007) found that both first generation Mexican immigrant parents and their children in the United States perceived that parental discipline became more severe after migration, often causing conflict between adolescents and their parents. The authors note that severe discipline and vigilance is not a continuation of traditional parenting practices from the home country, but rather is a response to the new setting which demands new strategies of child rearing.

In this paper, we build upon this work, as our goal is to show how immigrant parents in the Netherlands rebuild their parenting postmigration and find new solutions that result from a struggle between considering varied answers to concrete issues and problems originating in culturally diverse parenting practices. We add to the literature by showing how particular contact zones work, by focusing on the particular configurations of immigrant parents’ social networks, and on how they use their networks to find solutions for parenting dilemmas. We demonstrate how these networked contact zones can vary greatly among parents, producing unique dilemmas and possibilities for the reconstruction of postmigration parenting. The study thus must be read as a further elaboration of the strand of studies on immigrant parenting and immigrant families that have argued against simplistic models of transformation and have claimed that qualitatively new practices develop in new contact zones. In this study, our goal is to show what forms these contact zones might take on and what might be the consequence for how parents develop solutions for parenting dilemma's.

In addition, we suggest that recognizing these parents as creative producers of their own solutions, and becoming conscious of their self‐made support networks and the resources they provide, can offer professionals and policy makers a new paradigm for the design of social services and support for immigrant parents.

### The Development of “Parental Capital” After Migration

In our definition of social network, we draw from Bourdieu's notion of “social capital” as the “contacts and group memberships which, through the accumulation of exchanges, obligations and shared identities, provide actual or potential support and access to valued resources” (Bourdieu, 1993, p. 143). In addition, we assume, as Levitt and Glick Schiller (2004) have argued, that these networks of relationships are not confined to national boundaries, and can take multiple different forms depending on the networking practices of immigrants. Although rebuilding parenting involves both parenting as practice and as the beliefs that parents hold about parenting, in this study, empirically our focus is on parental beliefs, or “parental ethnotheories” (Harkness & Super 2006). Parental ethnotheories refer to implicit or explicit ideas about the “natural” or “right” way to think and act regarding children, parenting and families, and are often linked to cultural themes that also operate in other domains of a culture (Harkness & Super, 2006). Thus, our goal is to show how the reconstruction of ethnotheories provides parents with new “valued resources” as Bourdieu calls it or to use another term by Bourdieu: new “cultural capital” that is closely intertwined with the specific configurations of immigrant parent's networks. The paper does not deal so much with ethnotheories “themselves,” but rather with what networks and resources parents have to reconstruct ethnotheories after migration. In line with Bourdieu's definition of social capital, we introduce the term “parental capital” to indicate the whole of resources parents have at their disposal (both social networks and how these provide access to ethnotheories) to think and act as a parent. Thus, in Bourdieu's terminology “parental” capital consists of both the social networks and the valued resources these provide that help parents to form their own ethnotheories. Formal and informal support system surrounding parents have been found to be of great influence on parenting beliefs and practices (Cochran, 1993; MacPhee, Fritz, & Miller‐Heyl, 1996). In these social networks, members may exchange social norms for behavior, either through approval or criticism, and they may model each other's practices. We suggest that particular networked configurations create specific contact zones that impact the way parents are able to rebuild their parenting in the new country.

Parents migrating from communities in which parenting is seen as a shared responsibility of the (extended) family often maintain a homogeneous social network to support their parenting in the host country (MacPhee et al., 1996). Van den Berg (2007) found that first‐generation Moroccan‐Dutch immigrant mothers were mostly supported by family‐based networks, while friends and professionals were almost absent. Even transnational ties with kin living abroad (mainly in the country of origin) remain a source of support for immigrant parents (Reynolds, 2004; Ryan, 2009; Ryan, Sales, Tilki, & Siara, 2008; Van den Berg, 2007; Zheng, de Haan & Koops, 2019). These networks with similar others, usually referred to as “bonding capital” (Gittell & Vidal, 1998), offer clear advantages such as strong mutual support, a high level of trust, and the ability to share concerns with those who have similar ideas and values. In the literature, these relative homogeneous networks of immigrant parents have been evaluated mostly in a negative tone when it comes to parenting. For instance, it is argued that they allow the conservation of premigration heritages, and might therefore function as a buffer against influences or ideas from outside; or they exert pressure to conform to group norms and high levels of social control and obligations (MacPhee et al., 1996; Ryan, 2009). However, these traditional family structures are under great pressure after migration, particularly as younger and more highly educated generations of immigrants seek to escape these traditional networks (MacPhee et al., 1996).

Bridging capital (Gittell & Vidal, 1998) or establishing ties with “different others” can be important for gaining access to new information which otherwise would not have been available within the network. The exposure to a variety of different parenting practices and values may be effective for mothers living in postmigration. Being exposed to this variety might allow them to construct fluid cultural identities and help them move and position themselves between different cultural frames of reference (Reynolds, 2004). Looking for support from different others might be related to the duration of their stay. The longer immigrants live in the host country, the more likely they are to meet others who are similar to them in other ways than their ethnicity or immigrant status (Reynolds, 2004). For instance, Ryan (2007, 2009) found that Irish immigrant mothers in England established new local ties based on the shared experience of being mothers. When these women became mothers, they entered new social spaces like the school yard or the toddler group and thereby met new contacts through their children. At the same time, through new technologies, immigrant parents are less dependent on actual physical locations to be together and establish or maintain social ties with others, but rather on the connections they maintain and build across locations (Diminescu, 2008). These social network connections are not only essential for being in touch with far away contacts, they can also be considered also as paths of information, support, and so forth, and can be seen as “forms of linguistic and social capital” (Lam, 2014, p. 503).

### Moroccan Migrants in the Netherlands

In this study, we focus on the situation of Moroccan migrants in the Netherlands, whose parenting traditions sharply contrast with those in the guest country (see for instance Jonkers, 2003; Stevens, Vollebergh, Pels, & Crijnen, 2007).

In a review article of 2007 (Pels & de Haan, 2007), we have documented the research available on this group. We focused on the differences in child rearing values and practices between their home communities and those in the Netherlands, while considering their socialization practices after migration as cultural reproductions that represent both change and stability, resistance and adaptation. The review showed that, for instance, in most of the communities these families come from in Morocco, child rearing is not just a task of the nuclear family as in the Netherlands, but of a network of kinsmen, affines and even neighbors (Pels & de Haan, 2007). This means that in addition to the parents, patrilinear uncles, aunts, older siblings and neighbors can act as authority figures. Moreover, authority relationships in child rearing in these communities in Morocco are relatively strict compared to the more open, child‐centered and liberal approach in the Netherlands. A similar contrast is also reflected in the difference between on the one hand Dutch middle class values of upbringing, which center on (gradually) enhancing children's’ autonomy, and on the other hand the focus in these communities on conformity and the development of a moral sense. The focus on conformity is supposed to enable children to act in accordance with the laws and the rules of the family and Islamic community.

These and other contrasts, together with a loss of kin relatives due to migration, make reestablishing their parenting a challenging task. Also in more recent research (van Beurden & de Haan, 2019), we have shown how Dutch‐Moroccan parents are urged to redefine the meaning of their parenting after migration as they experience tensions between what is good, allowed, and socially appropriate here and there. Finding themselves between the more child‐centered ideologies in the Dutch society and the more hierarchical family structures in Morocco, they struggle to find answers to concrete dilemma's such as how much freedom and privileges children should be allowed, and what it means to be close to your child, while still maintaining an authority relationship.

In the present study, we explore the way in which mostly first‐generation Moroccan‐Dutch mothers construct and use their social networks to rebuild their ethnotheories—that is, how they form new “parental capital” through social networks that give them access to new ethnotheories. It is not our ambition to reveal the nature of their ethnotheories, but rather how these come into existence, and how we might start to explain their variety. We adopt the perspective of the parents themselves as we document how immigrant parents evaluate their networked relationships in order to address the challenging task of rebuilding their parenting post migration. The network methodology we use here is a useful tool to examine how the specific connections that parents employ impact the ways they are able to reconstruct their parental capital. This approach allows us to assess to what extent parents draw upon the values and practices of their traditional communities and when, how, and why they start to look for resources outside of these communities. In addition, mothers’ accounts of how they use these networks and resources offer insight into how they weigh, combine, or deal with contradictory resources, and how these finally might impact the solutions they adopt. The following research questions guided our analyses: How do first‐ and second‐generation Dutch‐Moroccan mothers living in the Netherlands use their social networks in order to create parental capital within a postmigration context? Can we understand their strategies from (their use of) the particular networked structures, such as the composition of their network, the network density, or the availability of particular sub clusters? Ultimately, in this study, we are interested in learning more about how social relationships in migration mediate the complex task of rebuilding parenting in migration.

## Methods

### Sample

At present, approximately 402,492 inhabitants of Moroccan descent live in the Netherlands, making them the second largest non‐Western group with an immigration background (CBS, 2019). Dutch‐Moroccan immigrants mainly came to the Netherlands around 1950 for economic reasons, which later resulted in family reunion and large second and third generations. The Dutch‐Moroccan mothers in the present study are mostly first‐generation mothers who came to the Netherlands to join their husbands and start a family. Participants for this study were recruited from the participants of a larger study on parental support in migrant grass‐roots organizations in eight cities in the Netherlands (see de Haan, de Winter, Koeman, Hofland, & Verseveld, 2013). For the current study, we invited only women from Dutch‐Moroccan background (thirty in total), who were a member of a migrant organization in which parental support was addressed, and who had at least one child to participate in an in‐depth ego network interview. In total, twenty‐five mothers agreed to participate, who were all first generation Dutch‐Moroccan mothers and active in migrant grass‐roots organizations. One interview has been excluded from the sample description and analyses because this mother consistently refused to speak in detail about specific contacts within her network. To our knowledge no statistics exists on the percentage of Dutch‐Moroccan parents that join migrant organizations for issues of parenting. However, it is documented that these migrant organizations are widespread, and that they are an important resource for the community to get help or information. These organizations are used instead of formal services as immigrant parents often lack trust or familiarity with formal services and feel that formal services do not meet their needs (Ponzoni, 2015). According to Ponzoni (2015) members of such organizations are generally the ones that are aware of specific issues and problems on parenting in their community and stimulate discussions on these issues. Parents who join such grass‐root organizations are generally seen as representatives of their community, who are able to voice issues for their community. In some respects these mothers vary from the general population of first generation immigrants, while not in others. In terms of length of stay, these mothers can be called representative for their (age) group. At the moment the interview was held the participants were on average 20 years in the Netherlands, whereas the average length of stay of the first generation of Moroccan immigrant parents aged between 25–50 years is 19 year (CBS, 2011). Our sample is however higher educated in comparison to the average first generation immigrant parent in the Netherlands. While in our sample 31% has enjoyed higher education, in the average population of first generation immigrant parents from Morocco this is only 5% (CBS, 2006). Thus, most likely due to the fact that we are sampling from active members of grass‐root organisations, our sample is higher educated in comparison to the average first generation parent. However, given that these parents are in close contact with the circles of their own ethnic community, it can be expected their ideas reflect important knowledge on parenting in their community.

### Procedures

In‐depth ego network interviews were administered from May until September 2011 by the second author and two trained master graduate students. The interview lasted on average 1.5 hours and consisted of three different parts. Informed consent was obtained and participants received a voucher for their participation which could be exchanged for goods in most stores in the Netherlands. In part I the mother was asked to express her vision on parenting, potential changes her parenting had undergone through the years as well as potential factors that might have influenced these changes. In part II we generated the network of the mother by asking the mother to list the names of the people that were in one way or another a resource for their parenting. We prompted the mothers to generate a list of important relationships with people they talk with, either superficially or in depth, about their children and/or parenting beliefs and practices. Background information about all contacts was gathered (age, gender, ethnicity, location, type of relation, and years the mother knew the contact), and this information was entered in NodeXL software. Mothers also indicated who of their contacts knew each other, in order to establish the density of their networks. A visual representation of their network generated by NodeXL served as input for Part III. The software automatically generated sub clusters within the network based on the information of who knew who. In more technical terms, the (clustered) position of alters, as related to each other and the respondent, was determined using the Harel–Koren Fast Multiscale algorithm, which is one of NodeXL's force‐directed algorithms. That means that the contacts, called alters, naturally push away from each other, while edges, that is relations expressed by connecting lines, bring them closer together. This results in highly connected nodes migrating to the center, while less connected nodes are pushed to the outside. The “groups” function of NodeXL was then used to calculate clusters, which works by aggregating closely interconnected groups of nodes.

Mothers were interviewed on how they use their network to solve important issues for their parenting, asking questions as: “To whom of all people within your network would you go to if you have a question about parenting?,” “From whom did you learn something about parenting and what was this?” Mothers were asked to identify the functions of the different relationships and clusters in the network picture. For instance, mothers were asked how the family cluster functioned for their parenting in relation to what the function of professionals or colleagues was. All participants were able to speak sufficient Dutch to participate in the interview, with one exception where the oldest daughter of the mother sometimes had to assist with the translation. All interviews were audio‐recorded with consent from the participants, except for one (which was written down by hand and proved to contain enough information to be included in the study).

### Analysis

The interviews were transcribed and the NodeXL data were all transported into SPSS. Discourse analysis was combined with quantitative data analyses. A discourse analytical perspective was applied in order to identify how the stories on parenting of the participants might not only represent their ideas, but how these ideas represent their practices of finding out how to juggle between the sometimes contradictory resources they found in their social circles. We are making use of a discourse analytical perspective as represented by, for instance, Gee (2018) that builds upon the vision that the stories people tell represent as well as recontextualise, and therefor thus in part make visible, the social practices they are part of. The quantitative network analyses focused on the structural and compositional characteristics of the networks. Using ANOVA the groups that were distinguished in the qualitative analyses were compared regarding personal network characteristics and a number of personal background characteristics of the mothers, see Table 8.1 for details. The discourse analysis focused on how mothers saw their social networks, and other resources they mentioned as related to how they solved particular parenting issues, as well as on how these solutions represented a reconsideration of their ethnotheories. Questions for this analysis were: *“When and how do mothers consult others, their network with regard to parenting?”* and “*How can we understand their use of their network in relation to their possibilities to rebuild their ethnotheories or new parental capital?”*

**Table 8.1:** Participants’ Background Information and Network Measures

	Total	Mean Independent Parents,	Mean Navigating Parents,	Mean Selective Parents,	F/Sign Oneway Anova
	n = 24	n = 5	n = 9	n = 10	
Age	36.4	35.6	35.2	37.9	n.s.
Educational level^a^	2.9	2.6	3	3	n.s.
Years of residence	20.1	20	17.1	22.8	n.s.
Number of children	3.0	3.2	3.0	3.0	n.s.
Network size	14.3	13.6	15.4	13.5	n.s.
Proportion family members relative to network size	.46	.53	.48	.39	n.s.
Proportion same‐ethnic members relative to network size	.64	.78	.70	.53	6.713/*p* < .006^b^
Number of other ethnic members in network	5.04	3.0	4.78	6.03	3.779/*p* < .004^b^
Proportion professionals relative to network size^c^	.16	.15	.20	.14	n.s.
Network density	.57	.65	.56	.53	n.s.
Proportion of transnational (excl. EU^d^)	.14	.88	.17	.15	n.s.

a Educational level is measured on a 0–4 range with 0 no education, 1 primary education, 2 secondary education, 3 vocational education and/or extra training and 4 higher education/university.

b BonFerroni post hoc test shows that in particularly the specialized parents differ from the other two groups for the proportion people from the same ethnicity in the network (*p* < .007) and for the number of people with another ethnicity (*p* < .039).

c Professional affiliation is measured on a 0–2 range with 0 no work, 1 voluntary work and 2 paid work.

d Network ties with contacts (*n* = 11) living inside the European Union were not considered transnational because they lived in near proximity (max. 3 hours) from the mothers, for example, Germany/Belgium.

## Results

We found that three discursive strategies could be distinguished based on the discourse of the mothers, including their interpretation of their network pictures. Although the three types were established “bottom‐up” from our data, we consider them to represent the various ways in which these mothers were able to operate in these so called “contact zones,” and deal with the conflicting value loaded resources their networks offer them. In order to confirm the reliability of the typology, two researchers not involved in the study but with knowledge about parenting, migration and social network analysis were asked to assign the cases to one of the three types. This procedure confirmed the typology that was originally found. Although our analyses are primarily based on the mothers’ discourse, we also investigated whether the mothers who belonged to a different “type” also differed with respect to network structural characteristics (network size, density, ethnic homogeneity and representation of family members and professionals); or mothers’ background characteristics (years of residence, educational level and work status).

Below, we describe three different ways in which mothers used their social networks in order to solve particular issues they came across in their parenting. We term them the navigating mother, the independent mother, and the selective mother, referring to how they related in their parenting to their traditional community.

### The Navigating Mother

This group of mothers is characterized by their active search for resources in both their traditional community and a newly formed community, consisting of professionals or neighbors and friends with a different ethnic background. They take both these communities as authoritative resources when they need support. As a consequence, they constantly “navigate” between sometimes contradictory cultural positions. We choose the term navigating to express both the need to steer due to being in an environment that is sometimes confusing, but also to express the fact that these mothers took agency to overcome contradictions. They presented themselves as active learners in the area of parenting, stepping out of their “comfort zone,” keeping their eyes and ears open for new knowledge, seemingly defined by a deeply felt need to do things differently, and to know how to act in the new situation.

The following excerpt is an example of this attitude, in which the mother states that she needed to step out of her “circle” to learn new things:

**Excerpt 1 “Seeking support”—Mother, 45 Years**

*Mother: Before, I did not have contact with many people. And you always stay in the same [draws a circle on the table]*.

*[…]*

*Mother: If you often, if you for example if you go away from the circle and you hear what is new for you, you are always surprised. Huh? What is this? How should you do this?*

These mothers, as the mother in the example above, actively created a large network to support their parenting in order to gain enough information. The difficulty they experienced was to combine the advice and information they were obtaining from, on the one hand, their family based network, and on the other, their newly established contacts. This sometimes resulted in indecisiveness, as the following example illustrates:

**Excerpt 2 “Indecisiveness”—Mother, 32 Years**

*Mother: So I am caught up with it in the sense of “Am I doing this right?” And then I read something from the CJG [Dutch Local Health Care Centre] and then I think “Oh, I'm not doing this right,” and then I read something from the baby clinic and I think “Well, I almost did this right.” And from my Moroccan culture, I actually did not do anything right. Then there is an aunt who says: “No, you have done a great job. If he wants a candy, you should give him one, that's good, it will keep him satisfied.” And the baby center (says): “Only one candy and then let him cry.” This [crying] is good for his lungs, they say. And at home they say, “That is not good for the neighbors.” So what should I do? I tell you honestly, I still find it very difficult*.

**Figure 8.1:**
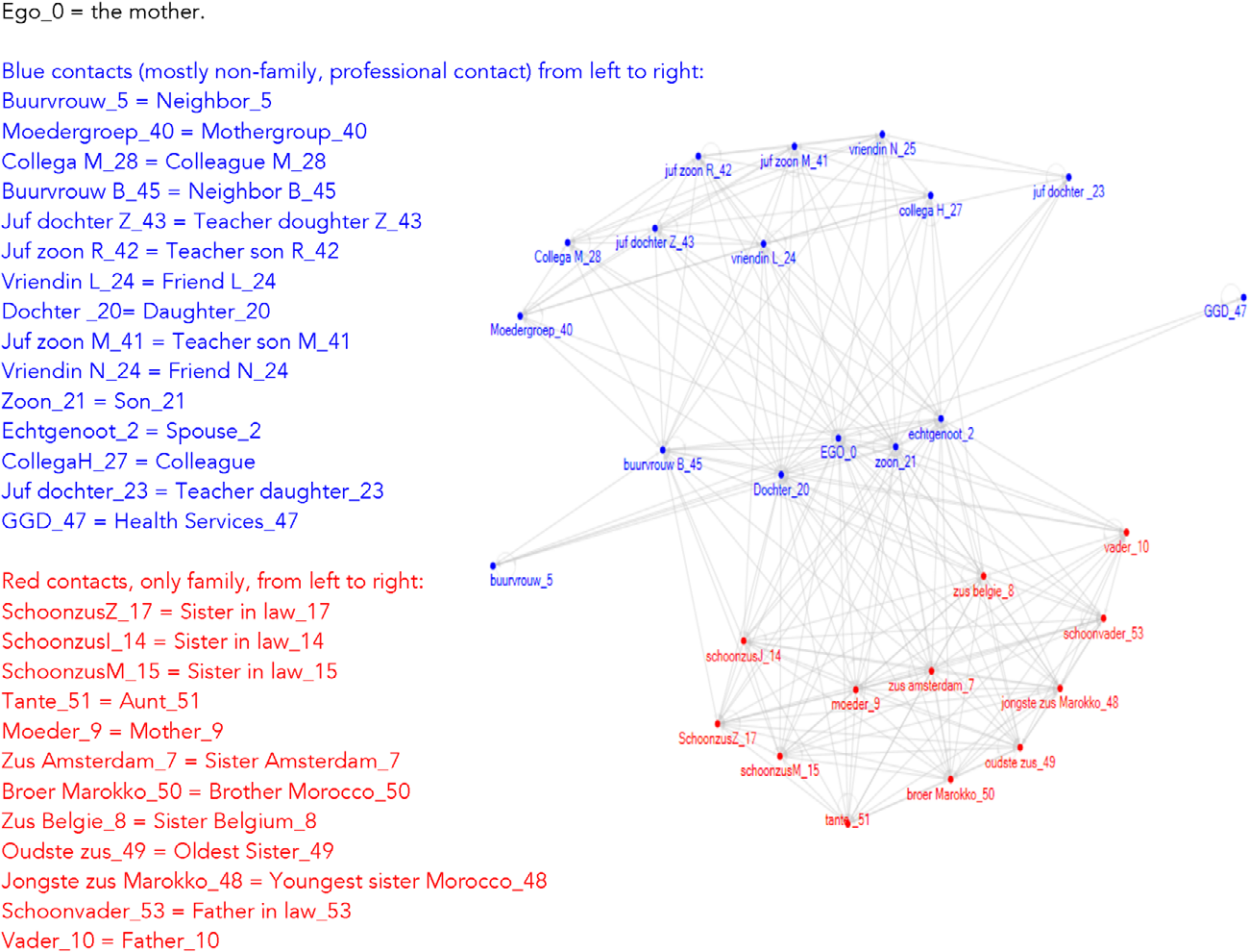
Example of a network of a Navigating Mother with two independent sub‐clusters.

Apart from indecisiveness, these mothers sometimes experienced incompatibility among different systems of parenting. For instance, mothers sometimes experienced values from a child‐centered parenting ideology, such as having an open and confidential relationship with their child, as contradictory to being an authority figure who has to set the rules. The fact that they lived and moved between different worlds was also clear in how they experienced their social networks. They identified their family network sub‐cluster as clearly different from “the rest” of their network, which often consisted of professionals, Dutch neighbors, or friends with differing ethnic background. An example of such a network is shown in Figure 8.1, in which two separate sub‐clusters are shown: one family‐based cluster, and one relatively unconnected cluster consisting of a mix of diverse contacts, including Dutch professionals and other contacts of Dutch descent. These two sub clusters in their network correspond with the two different authoritative sub communities that provide them with sometimes contradictory messages on what is the right thing to do.

### The Independent Mother

Mothers in this group were cautious with respect to who they trusted as a support provider in their parenting network, as they experienced the parenting beliefs and practices of their family network as dissimilar to their own parenting ideas. They believed that they had already learned more, and were critical adopters of new knowledge on parenting, striving to control their own decision‐making. In principle, they knew they could rely on a dense family‐ based network. However, with respect to parenting issues, they neglected this resource, which they described as forced upon them. They described themselves as self‐sufficient and independent in matters of parenting, and when they did seek help, they found it mainly from external sources like the internet, media and books, and a few isolated contacts.

**Figure 8.2:**
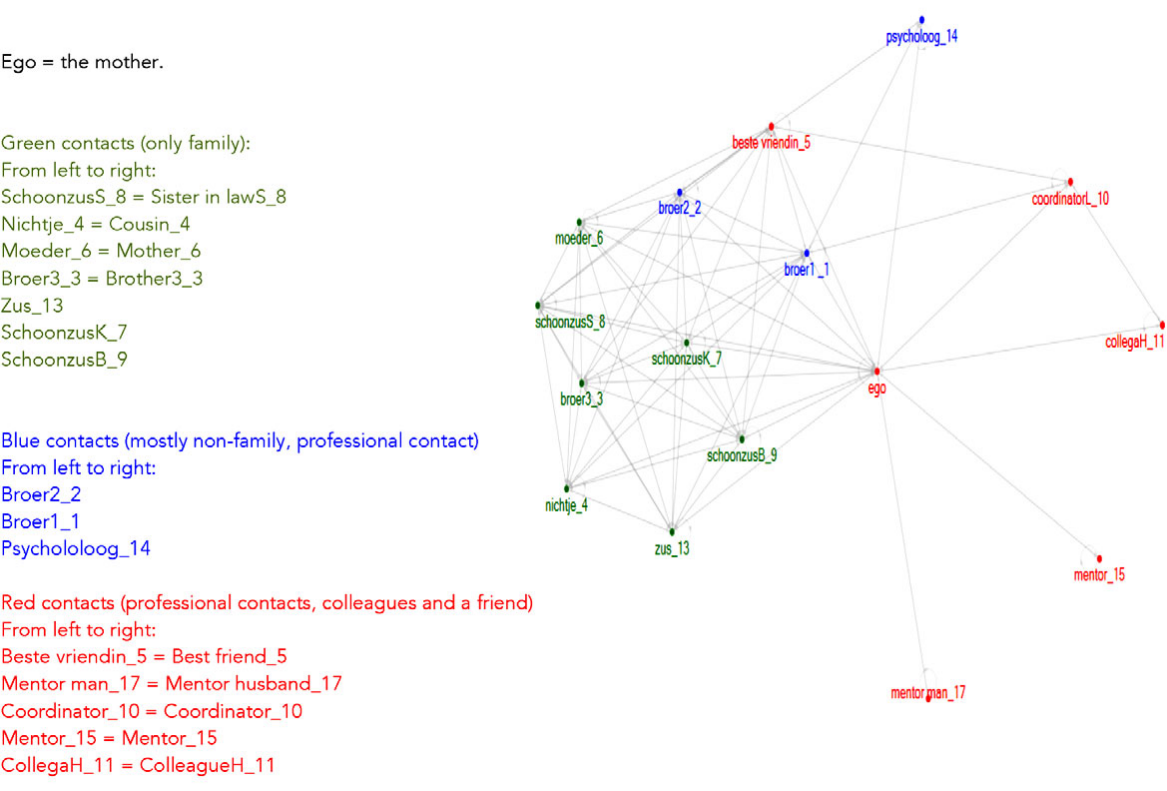
Example of a network of an Independent Mother who is reluctant to use the family‐based network.

Such a network is illustrated in Figure 8.2. A dense family‐based network is found next to a few colleagues and professionals who do not know each other. These mothers thus ignored the family based cluster, and relied on the self‐selected individual contacts. The difference between this type and the navigating mother, is that these mothers did not consult their family‐based network when they needed answers to questions of parenting that they struggled with. Also an alternative sub cluster that is relatively dense, as for instance present in Figure 8.1, is absent. Their negative evaluation of the support of their “own” ethnic group, and a withdrawal from this group when it came to strategic decision‐making in parenting is illustrated in the following example:

**Excerpt 3 “Use of External Sources”—Mother, 36 Years**

*I: And why do you ask the help of your niece? And why (do you not ask the help of) your brothers or mother, for example?*

*M: Because they have (..) their own parenting style and I do not agree with them*.

*I: Your brothers and your mother?*

*M: Yes. That is a conscious choice because then I don't have to mingle myself in unwanted discussions or debates*.

*I: Ok. Because you just said that sometimes your toddler has a terrible temper. What is something your brothers and mother would say then? What solution could that be?*

*M: For example you have to act stronger upon him, or you just have to punish him more often. And then I think (..) no*.

*[…]*

*I: And are there any others who you would turn to besides your niece?*

*M: No. Well the internet. That is really my handbook. When I miss something, then I just turn on the internet. To look things up or uh. Well and it makes me so wise you know. And there (online) I really feel like yes, I do not need anyone else*.

In this example, the mother rejects the harsh punishment of her toddler which she anticipates her family members (her mother and brother) will advise her to use when her toddler displays a bad temper. The mothers in this group preferred to turn to other resources, whether it be like‐minded others, such as the niece in this example, or the media. As this example shows, for this group, the internet served as a valuable, and anonymous resource that offered endless possibilities for learning about parenting, and allowed them to develop without having to justify their own parenting decisions to their kin.

### The Selective Mother

**Figure 8.3:**
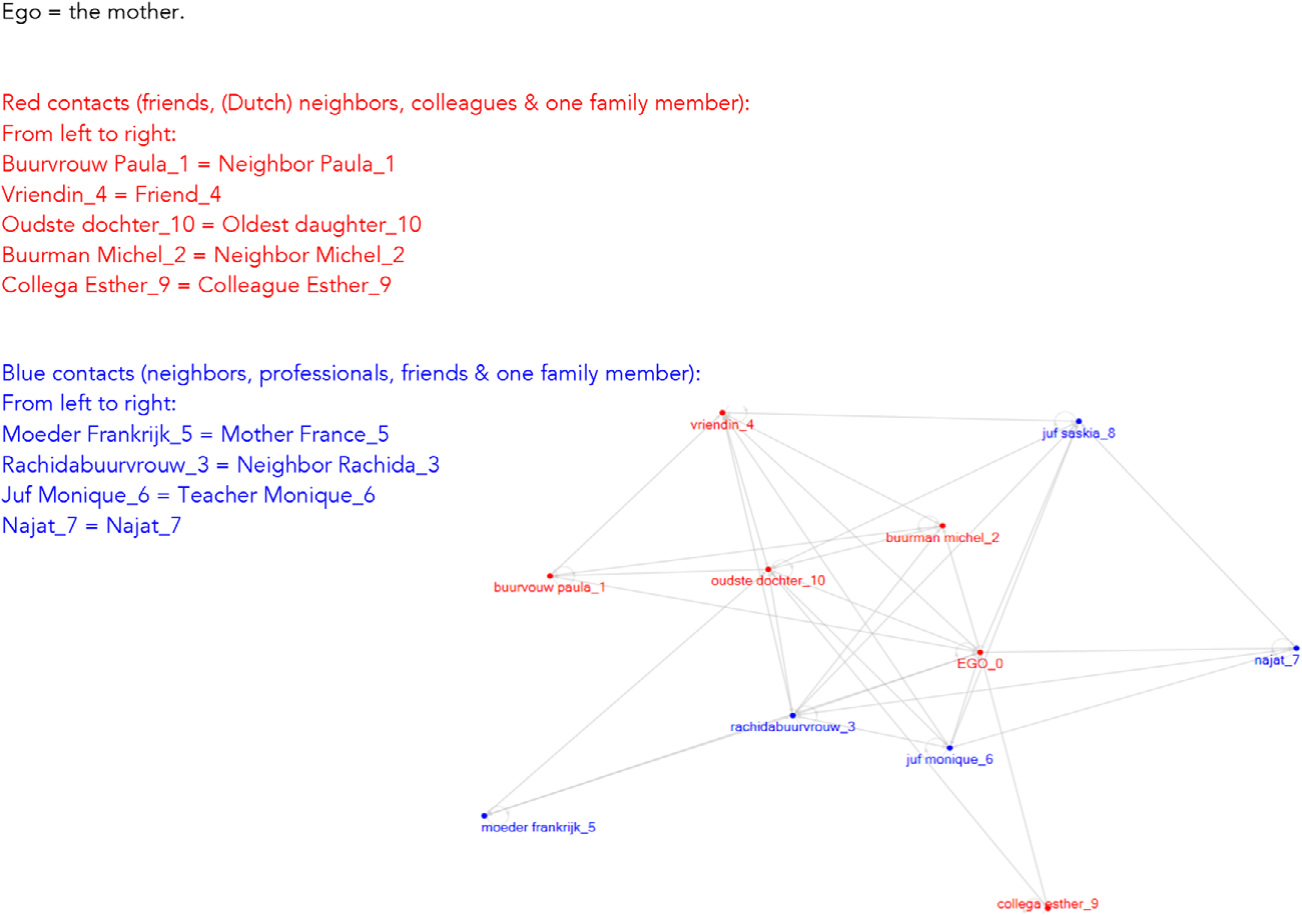
Example of a network of a Selective Mother.

This third group of mothers that we distinguished were very careful and selective in who they involved in their parenting. Like the Independent and the Navigating mothers, they were actively seeking support from others to gain new knowledge. They were unique, however, in that they built their own network without even considering the use of their family network. This is also clearly visible in Figure 8.3, which is an example of an independent mother's network. Characteristic is the absence of a family network and relatively unconnected other contacts.

These mothers seemed not to care about ethnicity when they asked for advice, as illustrated in the following excerpt where the interviewer is asking if the mother recognizes particular cultural parenting practices.

**Excerpt 4 “Parenting Is Not Ethnic”—Mother, 34 Years**

*I: What do you think is meant by Dutch parenting? What is typical Dutch?*

*M: Typical Dutch?*

*I: Yes*

*M: If you eat stamppot [local Dutch dish] this is typical Dutch*.

Laughter

*I: And what about parenting, is there something about which you think this is how people do it here?*

*M: No, parenting, I think parenting is all the same. I think uh yeah, that parenting is the same. If you are Dutch, Moroccan or Turkish*.

This group of mothers was most open to professional or scientific insights, which they saw as “in the best interest of the child,” as in the following excerpt in which a mother tells the interviewer that she is receiving a lot of criticisms from both Moroccan and Dutch mothers for the fact that she accepts professional help for her son:

**Excerpt 5 “Open to Professional Knowledge”—Mother, 34 Years**

*M: “And then they look at me as if they want to say “You as a Moroccan mother, you want this?” Then I say “Yes, it is in the best interest of my child, it is not about my interest” Look, I have a job, I have a house. But he still has to strive for this.”*

This is perhaps not surprising given the fact that this group was often more actively participating in paraprofessional settings, like parent committees, school boards or community centers where they come across professional knowledge when they collaborate with (Dutch) professionals. In addition, this group was most likely to be involved in paid work, and they also were the ones who were most actively involved in advising others on parenting. An illustration of this is the mother in the following excerpt, who advises her sister on a regular base on issues of parenting, as she feels her sister lacks knowledge in this field. This mother stresses the importance of change and making your parenting fit with “this time” as opposed to whether or not parenting fits with old or new traditions.

**Excerpt 6 “Sharing Parental Knowledge With Others to Do Things Differently”—Mother, 38 Years**

*M: You have them (children) now and you really should, from those books, you learn really a lot. (..) It is new, it is not like the past. Nowadays you have digital, (you have to) know about everything, lover boys, and uh yes these telephones, mobiles. Those are the things that I tell her (her sister), you should really do this differently. Unfortunately times have changed and you should be familiar with these things*.

With regard to the network characteristics, the three types differed significantly with respect to the number of relationships they involved in their parenting from their own ethnic community (relative to their network size), and consequently with respect to the total number of people they consulted from outside of the immigrant community. The mothers from the three types did not systematically differ on other network factors (network size, density, ethnic homogeneity, representation of family members, professionals, or transnational relations in their networks), nor did they differ in the background characteristics we measured: educational background, years of residence, age, family size (see Table 8.1). Rather, the distinction we found is based primarily on their discourse about how they used their networks, as well as on how diverse their networks were and how the sub‐cluster that represented their traditional community was positioned within the total parenting networks.

## Discussion

Contrary to the commonly held idea that parents either live in closed ethnically based networks or have entered new social spaces while giving up the bonds with their traditional communities, this study showed that parents are able to do both, and to apply diverse strategies to live among multiple worlds. Our main claim is not about how their networks look like after migration in general, but rather points to the fact that parents are able to rebuild their networks in a variety of different ways, and that this impacts upon, and is reflective of how they build their ethnotheories. For instance, being in touch with a more traditionally oriented community does not automatically imply that knowledge‐building practices concur with the values and practices of that community. This study shows that parents are resourceful in dealing with the diverse values and practices they encounter in their environment and deal with the potential incompatibility of these values and practices in various different ways. These strategies might very well have an impact on the kind of solutions these mothers acquire. In any case they seem to demand a different kind of effort from these mothers to establish working solutions for their parenting. Interestingly, the Navigators experienced the highest pressure of incompatible cultural systems as they considered both their traditional community and their newly acquired network in the Netherlands as serious partners in their attempt to create workable solutions for parenting in the new setting. They actively brought these conflicting solutions together, and worked hard to translate one system into the other. However, they did not experience themselves as highly efficient parents because of the tension this brought. The Independent mothers were active network constructors, trying to avoid certain influences, as they already knew that these solutions did not work for them. These mothers experienced themselves as “different.” Although they searched for input from like‐minded others, or information for their parenting outside of their communities, they are nevertheless in close touch with them. Selective parents spoke as if they were beyond the contradictions of the different parenting traditions, and stressed that parents need to adapt to the new situation, and that change is necessary. They were conscious network users, and also saw themselves as providers of new information and solutions for their peers.

In terms of the role our methodology played in our findings, we suggest that the mixed methods approach, combining network and discourse analysis focused on network strategies, enabled us to reveal how the construction of parental capital is informed by the specific network structures immigrants find themselves in. The form these networked configurations take, and how they are employed by parents, gives us insight into the existence of particular contact zones between sub‐communities, or the lack of access to particular resources.

In other work, we have termed the specific forms networks can take as “networked configurations for learning,” pointing to how these networks can be conceptualized as culturally and socially formed places for constructing knowledge, that are shaped by the specific histories of mobility and contact zones of these groups (de Haan, Leander, Ünlüsoy, & Prinsen, 2014). In this study, we have shown how particular elements of networked configurations, in particular the heterogeneity of the network contacts, as well as the position of the traditional network in the network as a whole, go together with particular uptakes of how to deal with conflicting parental ideologies. We do not suggest that one causes the other, but, in line with social capital theory, which explains the mutual relationship between the construction of social network structures and agency, as well as with social network studies in migration (see, e.g., Ryan et al., 2008), we assume that these particular strategies generate a specific kind of social network on the one hand, and that particular social networks enable also certain strategies.

The typology we found in the present study is not a blueprint for Moroccan or other immigrant mothers worldwide, as establishing and maintaining a network is a dynamic process evolving from a continuous interaction between the individual and its changing context. Nor do we claim that the network structures and characteristics we found are representative for all immigrant parents or for relatively schooled immigrant parents.

Nevertheless, the typology that we identified for the present study is useful in exploring possible pathways that Moroccan mothers engage in after migration; and to some extent, these patterns might be relevant for immigrant parents elsewhere. Moreover, the typology is useful in pointing out the variety of ways in which immigrants can deal with culturally conflicting value systems. This might be the case in particular for immigrant groups that experience a relative large distance between ethnotheories in their communities of origin, and those in the country of settlement. Thus in this sense, the findings of the paper apply to the transformations of other immigrant populations worldwide.

As noted in our introduction, previous research on how migrant mothers establish parental capital in their host country has been mainly described in terms of acculturation or assimilation, suggesting that the creation of capital entails either an addition to or subtraction from one or the other culture. Typically, network studies primarily have documented immigrant parents as being supported by a family based network, which cuts them off from the rest of society (MacPhee et al., 1996; van den Berg, 2007). Even though some studies have described how over time, parents are exposed to external influences and develop identities, which allow them to position and move between different cultural frames of reference (Reynolds, 2004), this element is largely neglected. The present study adds especially to this last strand of research by opening up the “black box” of acculturation. Instead of defining which category immigrant parents belong to in terms of their adaptation process, we have focused here on the multiple ways in which their “in betweenness” is visible in their networked configurations. In line with Levitt and Glick Schiller's (2004) concept of transnational fields, our study confirms that these networks are formed through the “in between” social networking practices of immigrants. We suggest that the diversity of these networks also creates particular challenges and opportunities for the formation of new knowledge and information about parenting.

### Limitations

In this study, we were limited in detecting how the networking strategies of these three groups might be related to relevant background factors due to the small scale. We did find a relationship between networking strategies and key factors such as the ethnic variety in their networks as well as the presence and the use of a dense family network. We think a larger scale study with the same set up is needed in order to investigate the relation with more general background factors such as length of stay or education.

Also, due to the small sample, we were not able to describe how exactly ethnotheories might differ as a result of particular networking strategies, even if we do offer several suggestions in this direction. This might be possible in a larger study in which researchers focus on particular elements of both social networks and ethnotheories. A more qualitative uptake of this line of research is to look more closely at the ethnotheories of these mothers while focusing on the question of to what extent these ethnotheories have a hybrid character and represent “new” parental capital. Some useful elements of such an approach might be found in de Haan (2011).

### Implications for Professionals, Policy, and Program Developers

The focus on “in betweenness,” the fact that immigrant parents are creative and agentic producers of new parenting capital, and the fact that their solutions and new capital are also highly varied, is seldom taken into account by social services and parenting programs directed at this group. We think that the following elements should be considered.

#### Approach Parents as Agentic Learners

The present study showed that immigrant parents are active seekers of solutions, and they have by necessity already started to build their parenting theories and practices before they come in touch with social services related to parenting. An often‐heard complaint is that these parents feel their traditions as well as new solutions are not taken seriously, especially when these do not meet the criteria of mainstream parenting. We suggest that it is important to not take mainstream conceptions of parenting as a starting point for delivering services, but instead the learning process of individual or communities of parents. In such a way social services directed at this group may be able to use the potential of creative energy that comes from the discovery of finding new solutions, the awareness of being a learner and creator of new solutions (see also Hyeeun & Nelson Agee, 2018).

#### The Role of Professionals

Our study has shown how professionals play key roles for this group of parents in considering alternative norms and concepts of parenting. In many cases, professionals along with media‐based resources come to fulfill the role of the traditional community network. When professionals are aware of the transitional processes these parents are experiencing, they can direct their services and even the communication of mainstream or standard solutions toward supporting it.

#### Reconsider Mainstream Programs and Consider Bottom‐Up Parenting Programs

The fact that there is a large variety in the way parents rebuild their parenting, as well as that they make active use of a variety of cultural resources, implies that parenting programs and services that take mainstream practices as their point of reference will not meet their needs. Customized solutions that take into account the specific history of particular parents and parent groups are needed, while also acknowledging the tensions that are brought about by confronting different traditions of parenting. Alternatively, in designing services and programs, policy makers could make use of “bottom‐up” parenting programs, in which parents' perspectives, experiences, and participation are perceived as core principles for support and learning, and parents are seen as learning resources for each other (van Beurden, de Haan, & Jongmans, 2018).

## Conclusions

The present study focused primarily on how parental capital is formed, in terms of how networked configurations can be used to form ethnotheories. Further research is needed to investigate the specific forms of hybridity that these different situations create, and their effects. Our research suggests however, that there are differences in the extent to which these hybrid settings create ethnotheories that are “ready to use.” All the mothers in our study engaged in combining different traditions, and in this sense, all were involved in the creation of hybrid parental ethnotheories. However, some ways of building hybrid capital seem to cost more stress and work than others, while they also seem to differ in the extent to which the solutions were considered as already functioning for them. For theories of acculturation, it is therefore important to consider the fact that the “product,” in this case, their new ethnotheories, that result of the efforts of immigrants to deal with the tensions of sometimes cultural incompatible resources, might depend also a great deal on parents own network strategies and creativity to deal with what these offer.

Our analysis also has implications for how we think of isolated communities or groups versus “well integrated” ones in the area of parenting and beyond. Immigrants, as this study shows, are resourceful in developing linkages to information, identities and people that are formative for their development. They are selective and strategic managers of their networks, and they develop a variety of strategies to both stay in touch with their communities of origin and create paths that lead to new development. We hope this study may contribute to an approach in which immigrant parenting is studied and analyzed while keeping the complexities of the formation of parental capital in mind, and which is also consequential for developing new approaches to policies, services and programs directed at immigrant parents.
